# Sialadenoma papilliferum: Bibliometric analysis

**DOI:** 10.4317/jced.56055

**Published:** 2019-09-01

**Authors:** Antonio-Jorge-Araújo de Vasconcelos II, Luana-Rafaela Gerber, Stanny-Hagath-Maciel Saraiva, Pâmela-Oliveira de Vasconcelos, Lioney-Nobre Cabral, Tiago-Novaes Pinheiro

**Affiliations:** 1 Master of Dental Sciences, concentration in Oral Pathology. Professor in the fields of oral pathology, stomatology, oral diagnosis, and cariology at the Amazonas State University; 2 Dental Surgeon, School of Dentistry, Amazonas State University, Manaus, Amazonas, Brazil; 3 Doctor in biotechnology, concentration in health. Professor in the fields of oral pathology, stomatology, oral diagnosis and cariology at the Amazonas State University; 4 Doctor in oral pathology. Adjunct professor of Oral Medicine and Oral Pathology at Amazonas State University, oral pathologist, coordinator of the Service of Pathological Anatomy of the Amazonas State University

## Abstract

**Background:**

*Sialadenoma papilliferum* is a benign rare condition of salivary glands showing a characteristic papillary growth of the ductal epithelium that ends up being confused with more frequent lesions of the oral cavity. Objectives: To perform a bibliometric analysis of all articles about *Sialadenoma papilliferum* in the oral cavity and to add a singular case report of Sialadenoma in the lower lip.

**Material and Methods:**

A total of 36 publications referring to *Sialadenoma papilliferum* in the oral cavity from the PubMed platform was reviewed. The specific data were collected, and a bibliometric analysis was performed using Microsoft Excel. The results obtained were then compared with this new case report.

**Results:**

The people most affected with sialadenoma were white males at the average age of 56. The lesion was asymptomatic, usually white or red, with an average size of 1.4 cm, and the palate was by far the most affected site. The majority of the lesions were excised, and only two cases indicated recurrence.

**Conclusions:**

With surgical removal, *Sialadenoma papilliferum* has a favorable prognosis and no further treatment is required. Due to few recorded cases of recurrence, a long follow-up period is recommended to ensure that the lesion does not redevelop.

** Key words:**Sialadenoma papilliferum, salivary gland, oral cavity, bibliometric analysis.

## Introduction

Abrams and Finck first used the term *Sialadenoma papilliferum* (SP) in 1969 to describe two cases of an unusual neoplastic salivary gland proliferation that appears to be quite similar morphologically to the Syringadenoma papilliferum originating from the sweat glands (1). Therefore, SP is a distinctive rare benign lesion of the salivary glands (2), showing a characteristic papillary growth of ductal epithelium (3), and is classified as a ductal papilloma (4). SP’s origins have been controversial (5). Abrams and Finck believe that it arises from myoepithelial cells (1), but others have proposed that it is a result of focal hyperplasia and metaplastic phenomena of intercalated or excretory duct cells, following blockage of a salivary gland duct (6). SP clinical features can mimic other lesions, such as mucocele. It often involves the palate, lip, and buccal mucosa, and it usually affects old men and rarely children (7,8). Surgical excision is an adequate treatment for the lesion with a good prognosis because local recurrence is rare (9,10). The present article is intended to perform a bibliometric analysis of all articles in the Pubmed platform about *Sialadenoma papilliferum* in the oral cavity and a literature review and to add a singular case report of Sialadenoma occurring in the lower lip.

## Material and Methods

A bibliometric study of scientific literature was carried out with articles from the PubMed platform with the following descriptors: “*Sialadenoma papilliferum*,” “oral cavity,” and “case report.” A total of 65 articles were found, but those that did not concern the topic of interest, were not case reports, or included cases that did not occur in the oral cavity were excluded from the review. Consequently, 36 articles remained for the bibliometric analysis, which was performed using Microsoft Excel. The following indexes were taken into account: publishing country, patient race, age at diagnosis, patient gender, lesion occurrence site, lesion size, progression time, lesion color, symptoms, biopsy type, additional analysis, follow-up period, recurrence, and diagnostic hypothesis (data are given in Online Resource 1). Finally, the results obtained were compared with the case reported below.

**Figure 1:**
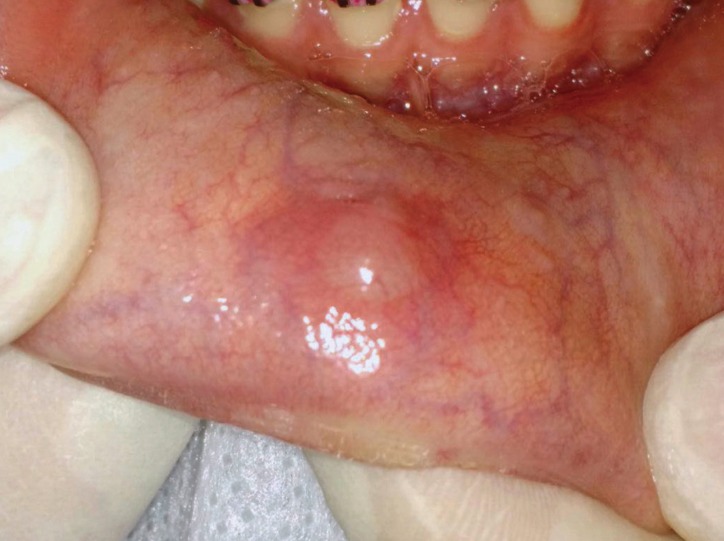
Nodular mass at the lower lip.

**Figure 2:**
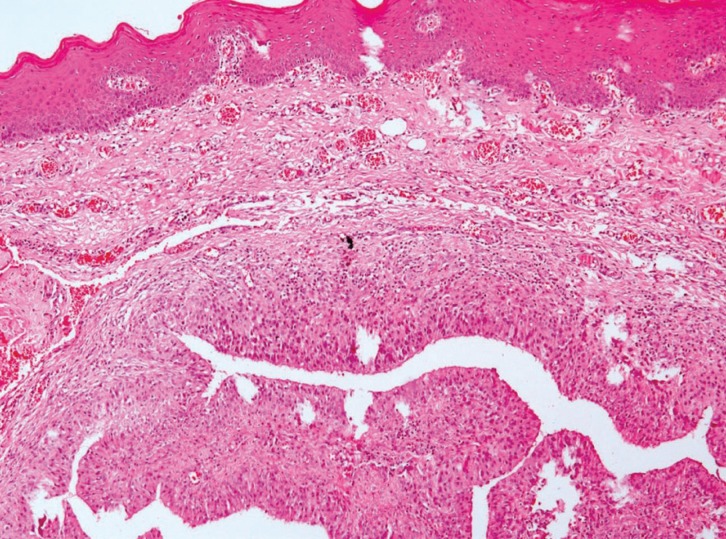
Exophytic papillary proliferation composed of stratified squamous epithelium and glandular proliferation occupying the submucosa (H&E, original magnification: 10X).

**Figure 3:**
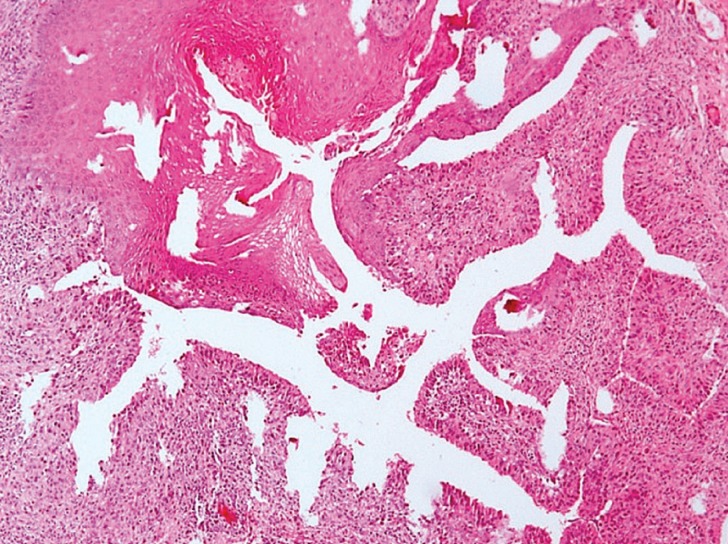
Ductal structures showing an irregular outline with infoldings of the glands (H&E, original magnification: 20X).

A 20-year-old female with no medical history presented with a 2 cm x 1 cm painless nodular mass on the lower lip with trauma due to an orthodontic treatment (Fig. 1). The tumor was excised under local anesthesia, with a suspected clinical diagnosis of mucocele, and submitted for histopathological examination. Histologically, the lesion showed an exophytic papillary proliferation composed of well-differentiated stratified squamous epithelium, which merged with a glandular proliferation occupying the submucosa (Fig. 2). The stratified squamous epithelium in the exophytic portion was hyperplastic with hyperkeratosis, parakeratosis, and focal hypergranulosis. The squamous epithelium also showed spongiosis. Ductal structures showed an irregular outline with infoldings of the glands (Fig. 3). The ductal epithelium was composed of luminal columnar cells with abundant cytoplasm and basally located round nuclei. The connective tissue showed chronic inflamation, and the minor salivary glands showed a normal appearance, along with the muscular and neural tissues. Based on these histological findings, the diagnosis of SP was established. After a 17-month follow-up period, the patient showed no evidence of recurrence.

## Results

The 36 articles selected for our review presented 50 SP case reports (1-5,7-9,11-37).

Publishing country – The country with the majority of publications was the United States of America (USA) with 18 (36%) cases. The second is Japan, which had one-third less cases than the USA with 6 (12%) cases. The complete distribution of cases per country is displayed on  Table 1.

**Table 1:**
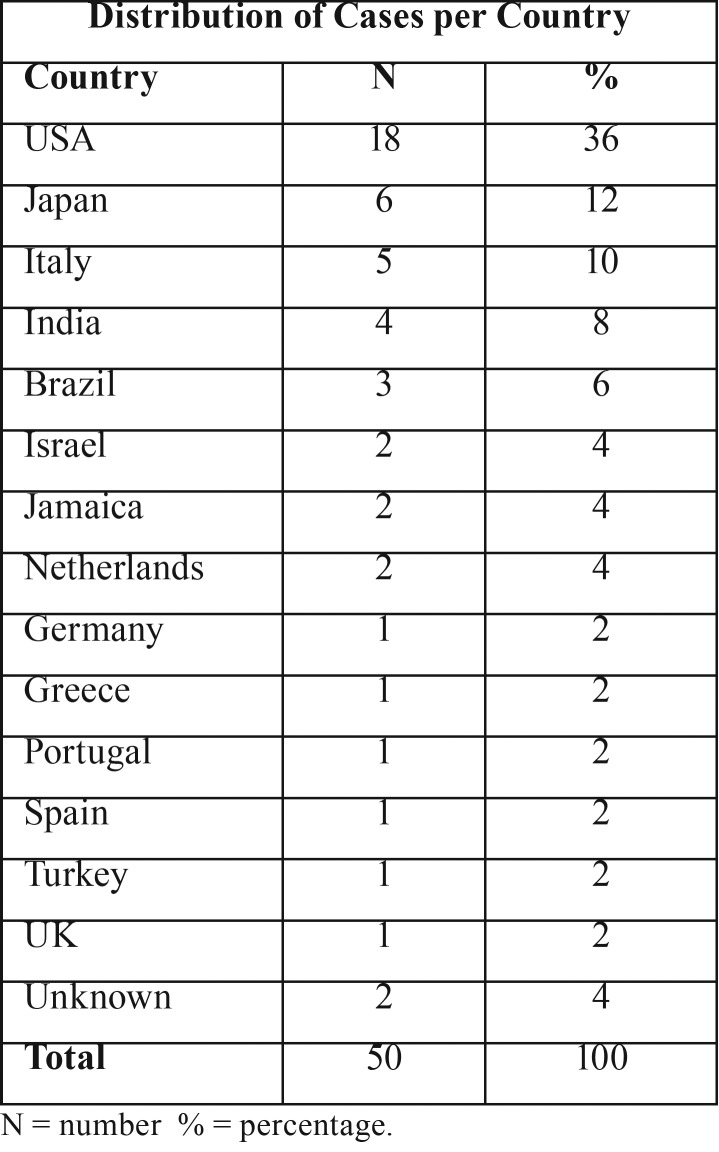
Distribution of cases per country. Based on literature review (1-5,7-9,11-37).

Patient race – Of the 50 reported patients, 18 (36%) were white, 5 (10%) were Japanese, and 3 (6%) were black. The articles included only 1 (2%) brown patient and only 1 (2%) Arabic patient. The remaining 22 cases (44%) did not state the patient’s race.

Age at diagnostic – All 50 articles provided information about the affected patients’ ages ( Table 2), showing that SP has been associated with every age group. The average patient age was 56 years, with a standard deviation of 16 years.

**Table 2:**
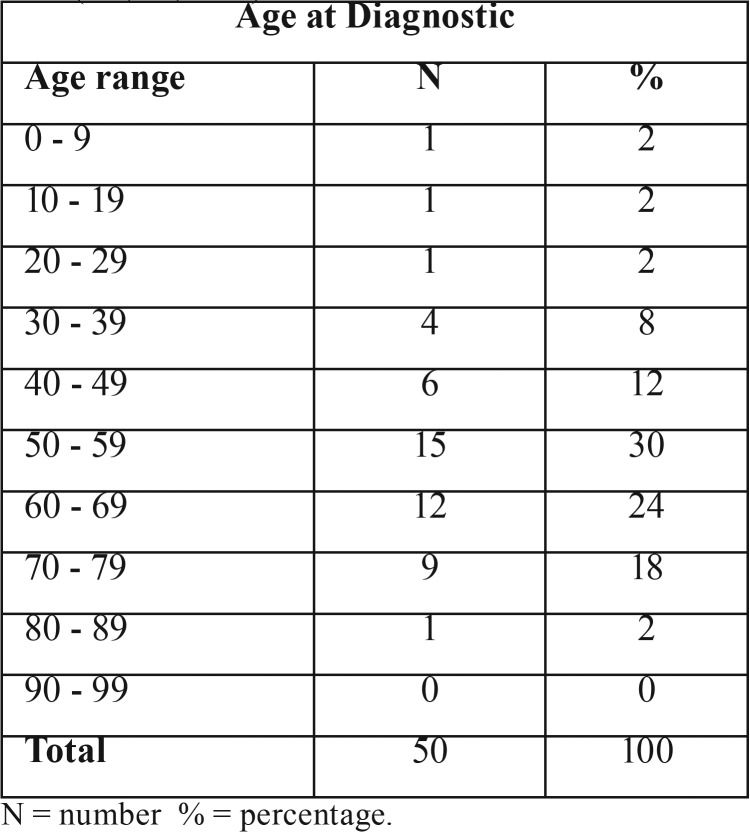
Age at diagnostic range. Based on literature review (1-5,7-9,11-37).

Patient gender – Of the 50 cases, 35 (70%) occurred in males, 14 (28%) were observed in females, and 1 (2%) patient’s gender was unknown.

Lesion occurrence site – The palate was the site with the highest incidence with 31 cases out of 50 (62%), followed by the buccal mucosa with 4 (8%) cases and the parotid gland, also with 4 (8%) cases. All reported occurrence sites are shown on  Table 3. Of the cases, 21 (42%) occurred on the hard palate, 6 (12%) at the junction of the hard and soft palates, 1 (2%) on the soft palate, and 3 (6%) of the cases did not specify in which part of the palate the lesion occurred.

**Table 3:**
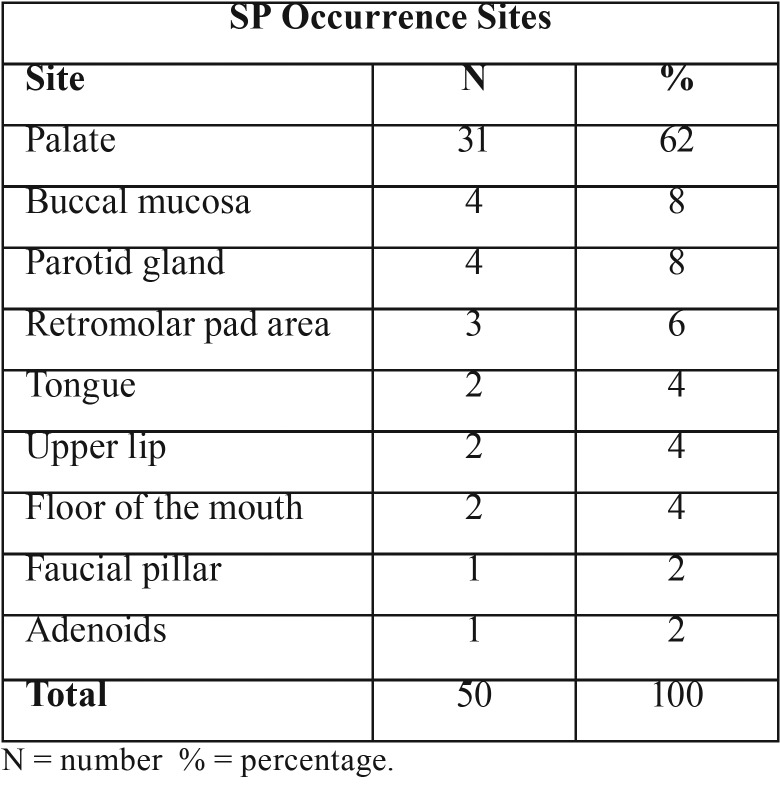
Sialadenoma occurrence sites. Based on literature review (1-5,7-9,11-37).

Lesion size – The average lesion size was 1.4 cm with a standard deviation of 1.5 cm. A large majority of the lesions (41 out of 50, 82%) measured until 2 cm in the largest extent, 3 (6%) ranged from 2.1 to 4 cm, and only 2 (4%) were larger than 4 cm. Of the articles, 4 (8%) did not include the lesion’s size.

Progression time – The average progression time was 52 months, with a standard deviation of 60 months.

Lesion color – The data showed that 5 out of 50 lesions (10%) were white, 5 (10%) were red, 5 (10%) were whitish velvet, and 4 (8%) were pink. Variances from these colors were also cited, as shown on  Table 4.

**Table 4:**
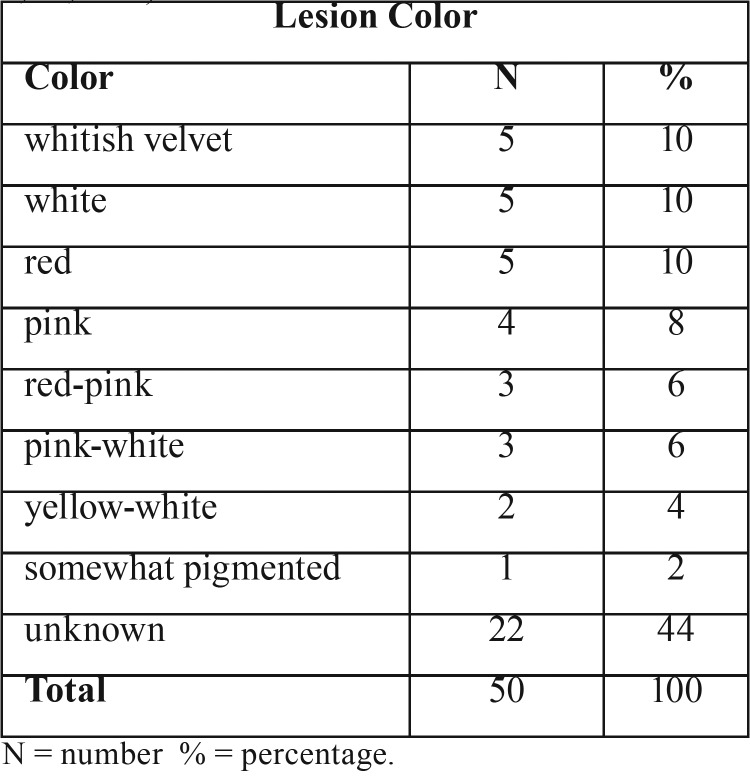
SP Lesion Color. Based on literature review (1-5,7-9,11-37).

Symptoms – In no case was the lesion reported as symptomatic. Of the 50 cases, 12 (24%) were recorded as asymptomatic, and 38 (76%) did not include any mention of symptoms.

Biopsy type – Of the 50 reviewed cases, 34 (68%) included excision of the lesion, 2 (4%) included incisional biopsy before the excisional one, and 1 (2%) included an autopsy of the lesion. The biopsy type was not indicated in 13 (26%) cases.

Additional analysis – Of the 36 articles, 7 included an immunohistochemistry (IHC) study, and 1 included a human papillomavirus (HPV) polymerase chain reaction (PCR) analysis. The 28 remaining articles did not include any analysis other than the histopathological examination.

Follow-up period – The average follow-up period was 23 months.

Recurrence – Only 2 cases out of 50 (4%) indicated recurrence of the SP lesion, 33 (66%) cases stated no recurrence, and 15 (30%) cases didn’t refer to this subject.

Diagnostic hypothesis – Of the 50 cases consulted, 36 diagnostic hypotheses were found. From the most to the least frequent: papilloma was the most mentioned, with 11 (31%) citations, followed by carcinoma with 6 (17%) citations; fibroma with 4 (11%) citations; and salivary gland neoplasm, also with 4 (11%) citations. Other diagnostic hypotheses mentioned were: Fibroepithelial with 3 (8%) citations, vascular lesion with 3 (8%) citations, mucocele with 2 (5%) citations, melanoma with 1 (3%) citation, verrucous leucoplakia with 1 (3%) citation and Warthin’s tumor with 1 (3%) citation.

## Discussion

SP is a rare neoplasm that accounts for less than 1% of salivary gland tumors and typically affects adults older than 50 (as seen on  Table 2) (38). The youngest patient, two years old, was reported by Sawyer in 1986 (34), and the fourth youngest patient with SP, to our knowledge, is presented in this paper. Our bibliometric study (Online Resource 1) found only three SP cases published in Brazil (T Table 1), and all of them occurred in adult patients (11,18), which makes this case the first one involving a young patient in Brazil. The reason adults are more frequently affected is uncertain.

Our case analysis revealed that males were more often affected by SP than females according to the literature and that more than one-third of the patients were white (3,4,16). Nonetheless, to our knowledge, no other article has included race prevalence.

SP appears as an exophytic papillary growth of the mucosa with color ranging from white to red ( Table 4) (3,4,16). In this case, though, SP manifests itself as a nodular mass without papillary features, similar to another case in the upper lip buccal mucosa (12). The present SP size matches the one reported in the literature, as it does not exceed 2 cm (16). The majority of the cases reviewed (41 out of 50, 82%) match this criteria; however, five cases included SP lesions bigger than 2 cm (1,13,14,31,37).

The literature states that SP often involves the palate, lip, and buccal mucosa (10). Based on the articles consulted, the palate was indeed the site with the highest incidence with 31 cases out of 50 ( Table 3). The buccal mucosa was the second most affected site but represented only 8% of the cases, and the lip had a low incidence of 4%. The present case was the only one reported in the lower lip. We still have no explanation of why the Sialadenoma occurs more often in the palate (20).

The SP is described in the literature as a typically slow growing lesion (13), a fact highlighted in our study, because the average progression time was 52 months. This narrow SP activity could be explained with low staining of Ki-67 (3), a nuclear protein that shows immunoreactivity when the cell cycle is active.

Because SP is a rare condition, it is hardly ever reported as a diagnostic hypothesis (as seen in our results) and ends up being confused with more frequently occurring lesions of the oral cavity, such as mucocele. Many of these lesions present themselves as small exophytic nodules and are asymptomatic like SP, which makes it impossible to conclude diagnosis only with clinical information. According to the literature, a differential diagnosis of SP should include a check for other benign salivary gland tumors, such as inverted ductal papilloma and intraductal papilloma, and hyperplasia (7,12). Besides this paper, only two other cases cited a hypothetical diagnosis of mucocele (10,12). One of them was also on the lip mucosa (12). That being said, we can assume that histopathological examination is essential to confirm an SP diagnosis and to rule out the possibility of other similar lesions.

To classify a lesion as SP in the histological analysis, we must identify superficial papillary projections that are lined by squamous epithelium and extend to submucosa to form cystic-like spaces (38). In the present case, the lesion met those criteria. It also presented acanthosis and parakeratosis as described in the literature (38). Some of our findings were also reported in other cases, such as chronic inflamation cells in connective tissue (3,10,12,30), epithelium with hyperkeratosis and parakeratosis (2,3,11,19,20), hyperplastic epithelium (2,19,26), epithelium with focal hypergranulosis (19), and spongiosis in squamous epithelium (19).

Besides the histological analysis, two additional examinations were found in our review: PCR analysis and IHC examination. The HPV PCR Analysis was performed in only one study, and no hybridization of any of the HPV-specific probes was found (29). The IHC studies were performed to determine SP’s histogenesis, which is still unclear (4,10,18). The following antibodies were used in these studies : AE1/AE3, carcinoembryonic antigen (CEA), epithelial membrane antigen (EMA), cytokeratin (CK) 7, CK13, CK14, CK19, Ki-67, P40, P63, vimentin, smooth muscle actin (SMA), and SM-100 (3,4,10,11,18,19,31). A normal salivary gland presents CKs 14, 13, 7, 8, and 19 (all of them found in the SP immunoprofile) but does not stain for vimentin or smooth muscle actin (18). The results for AE1/3, CEA, and EMA staining were also positive (19,31), yet the SP IHC exams for vimentin and SMA had different results among the cases, being positive in some and negative in others (3,10,18,19,31). These results could suggest that the tumor cells stem from various cell types such as duct cells and myoepithelial cells (3). Nonetheless, no article identified PCR or IHC examinations as routine parts of diagnoses.

With complete surgical removal, SP has a favorable prognosis, and in most cases no further treatment is required (10). According to Abrams and Finck, no evidence exists of disease 1½ years following surgery (1). Nevertheless, due to a few recorded cases of recurrence, a long follow-up period is recommended to ensure that the lesion does not redevelop (3). The follow-up interval and scope should be set individually. In the present case, a 17-month follow-up period showed satisfactory healing and no evidence of recurrence, similar to most of the consulted cases. However, in two cases in the oral cavity, the lesion recurred: with SP at the junction of the hard and soft palate after excisional biopsy, recurrence occurred at the 36-month follow-up (23); and with SP in the mucosal surface of the cheek after excisional biopsy, recurrence occurred at the 36-month follow-up (9). One of the consulted cases included a transoral robotic surgery, introducing a new technology to remove SP lesions, which provides good hemostasis, less pain, and limited surgical morbidity (37). The downsides of this treatment, though, are the high costs and uncertainties regarding the new technology (37).
